# Extended Reverse Dorsal Metacarpal Artery Flap: A Case Report

**DOI:** 10.7759/cureus.91736

**Published:** 2025-09-06

**Authors:** Luis Atilio Gil Perez, Erick Hidrogo Ordaz, Iberia María Ossa Nájera, Pablo A Mendoza, Diana Marisol González P

**Affiliations:** 1 Plastic Surgery, Hospital Real San José Valle Real, Guadalajara, MEX; 2 Plastic and Reconstructive Surgery, ISSSTE, Torreón, MEX; 3 General Surgery, Hospital General Dr Miguel Silva, Morelia, MEX; 4 Internal Medicine, ISSSTE, Durango, MEX

**Keywords:** dorsal metacarpal artery flaps, extended reverse metacarpal artery flap, extensor tendon exposure, local flap, middle phalanx injury, reverse dorsal metacarpal artery flap

## Abstract

We present the case of a 77-year-old male agricultural worker with poorly controlled type 2 diabetes mellitus (HbA1c 9.2%) who experienced a dorsal middle phalanx injury. He was otherwise healthy, with no history of hypertension, peripheral vascular disease, or coronary artery disease. The patient was a non-smoker and was not on anticoagulants, antiplatelet agents, or immunosuppressive drugs at the time of injury. He sustained a dorsal middle phalanx injury with extensor tendon exposure caused by a rotary grinder. An extended reverse-flow dorsal metacarpal artery (DMA) flap based on the second metacarpal vessel was performed immediately upon arrival at the emergency department. The patient showed favorable evolution, with minor flap loss and no need for further intervention. This report highlights the utility of this technique for achieving functional and aesthetic reconstruction in high-risk individuals.

## Introduction

Complex dorsal middle phalanx injuries with extensor tendon or bone exposure are challenging to cover and have traditionally been managed with advanced flaps, free flaps, or two-stage surgeries that often restrict limb mobility. Flaps based on the dorsal metacarpal artery (DMA) are an important resource for reconstructing digital defects [1]. One variation involves the extended metacarpal flap, which has been successfully used for phalangeal defects. It takes advantage of the consistent arterial connections between the DMAs and the palmar digital system at this level [2].

These reverse-flow flaps are employed for reconstructing digital injuries with joint, tendon (extensor), or bone exposure. However, the absence of certain branches of the DMA, as noted in cadaveric studies, should also be considered. It has been described that the first to fourth DMAs are the most consistent branches, while the fifth dorsal artery is present in 95% of specimens, varying only in its origin, with all these arteries having an approximate diameter of 0.5 mm [3-5]. We report a case involving the use of one of the branches of the metacarpal artery for the coverage of a dorsal middle phalanx defect with extensor tendon exposure.

## Case presentation

**Figure 1:**
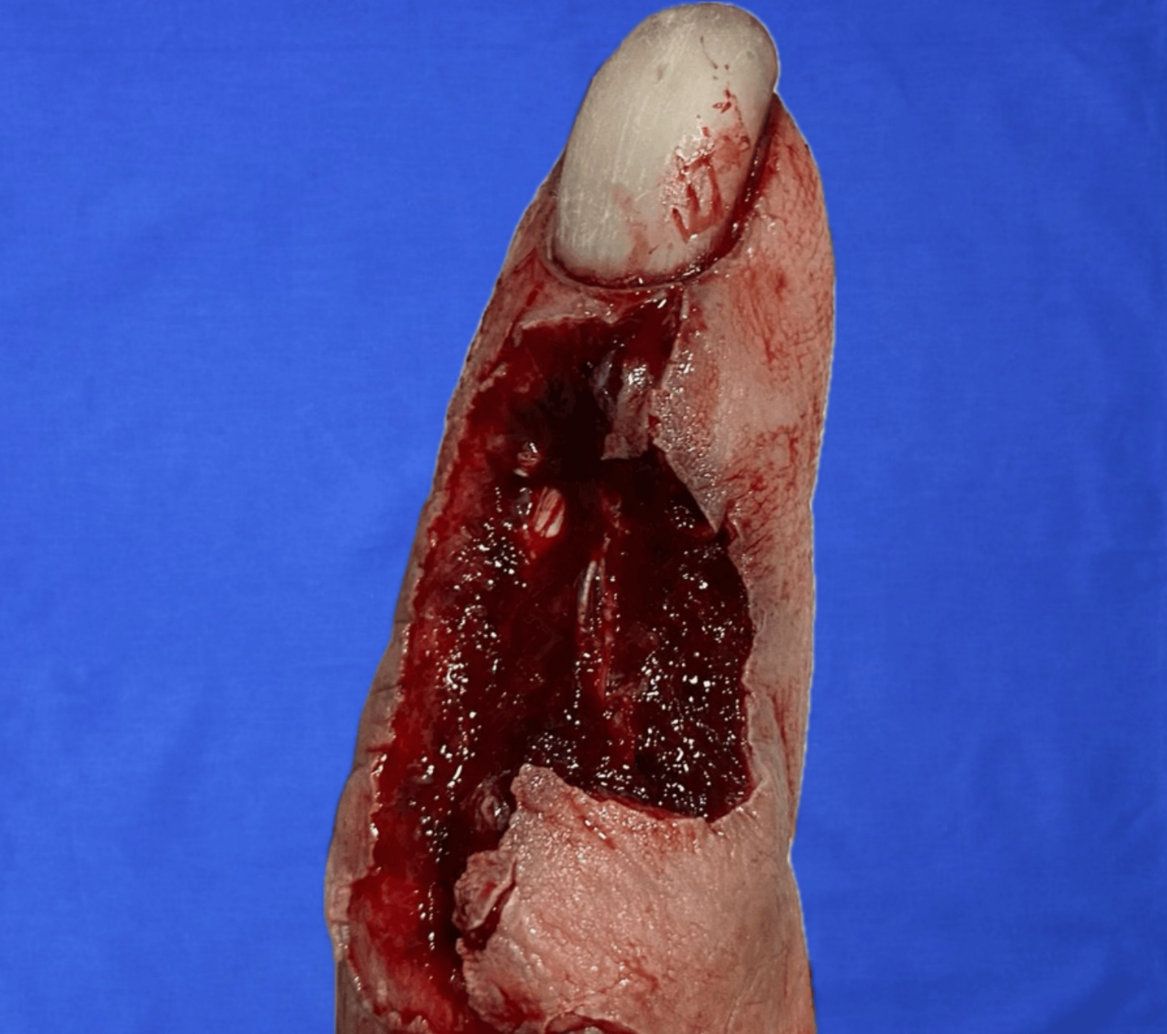
Dorsal middle phalanx injury Complex soft tissue defect of the dorsal middle phalanx with extensor tendon and bone exposure

A 77-year-old male manual laborer with a history of poorly controlled diabetes sustained a traumatic laceration from a grinder to the dorsal aspect of the middle phalanx of the right index finger two hours before presentation. On arrival, the patient had a 3.4 × 1.8 cm soft tissue defect with extensor tendon and bone exposure, irregular wound edges, and no active bleeding (Figure 1).

**Figure 2:**
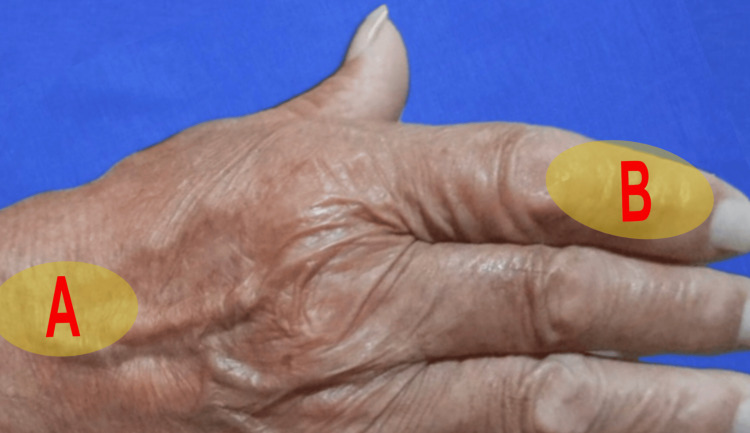
Preoperative planning Preoperative site (A) donor in the dorsal region of the hand and site (B) receptor zone

Given the patient’s comorbidities and the complexity of the injury, a reverse-flow DMA flap was planned and performed immediately upon admission to the emergency department. Preoperatively, the flap design was planned using the second dorsal metacarpal artery, with the donor site located in Zone A and the recipient site in Zone B. Given that the defect was located on the dorsal middle phalanx of the index finger, the pivot point was set at the junction of the base and middle third of the proximal phalanx of the index finger (Figure 2).

Surgical technique

After marking the donor area according to the required dimensions, a longitudinal incision was made on the dorsal aspect of the hand. The donor site was dissected layer by layer until the extensor tendons were exposed. The second DMA was identified, ligated, and divided at its origin to establish a reverse-flow pattern. The skin island was elevated in a subfascial plane, carefully including the vascular pedicle and its accompanying veins to preserve perfusion.

**Figure 3:**
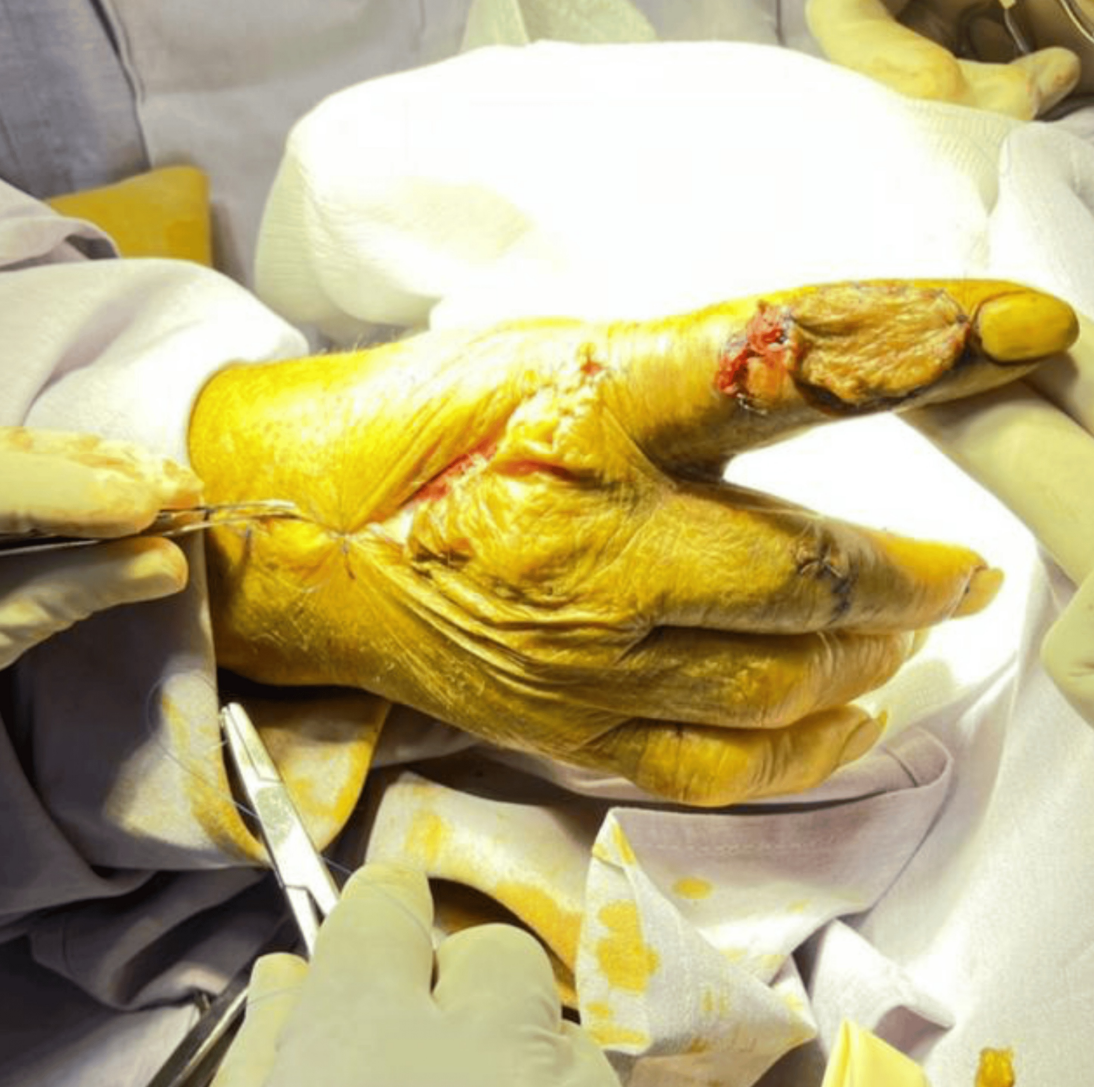
Transoperative image Transoperative image showing flap placed in the receiving site and with partial closure of the donor site

The distal connections between the DMA and the palmar digital arterial system were preserved, ensuring vascular inflow to the flap in a reverse-flow manner. The flap was then mobilized and tunneled distally to the recipient site. A skin bridge was maintained at the level of the middle phalanx of the index finger to facilitate flap transfer. Both the donor and recipient sites were closed with simple interrupted sutures (Figure 3).

Follow-up

**Figure 4:**
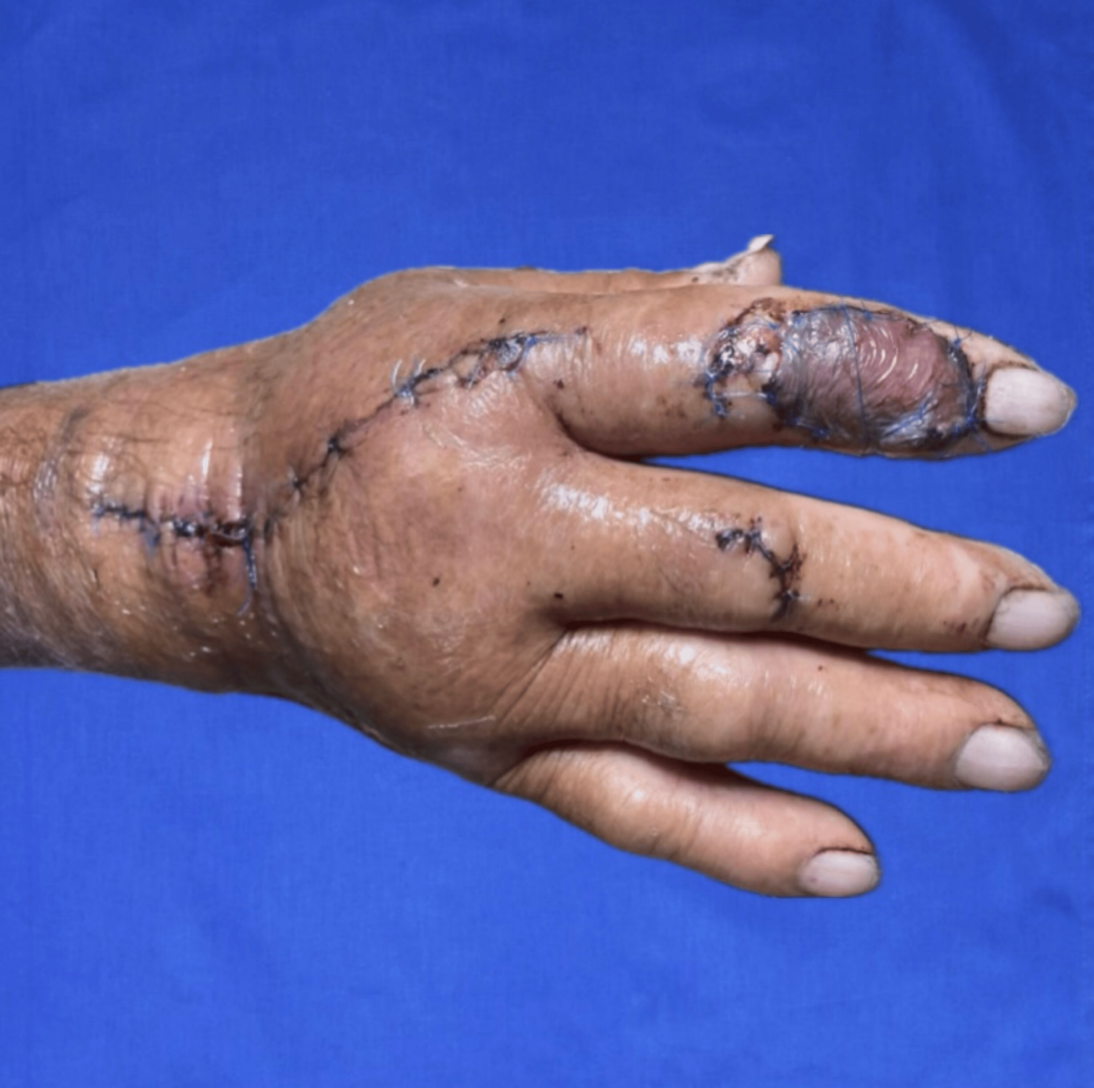
Follow-up image at 15 days Follow-up image after 15 days of the procedure, exhibiting venous congestion of 30%

The procedure was successfully performed. At two weeks postoperatively, the patient demonstrated good progress, with hand edema and venous congestion-related color changes at the recipient site. These were managed conservatively, resulting in partial flap loss of approximately 30% of the surface area (Figure 4). Despite this, the wound healed secondarily without the need for further surgical intervention.

**Figure 5:**
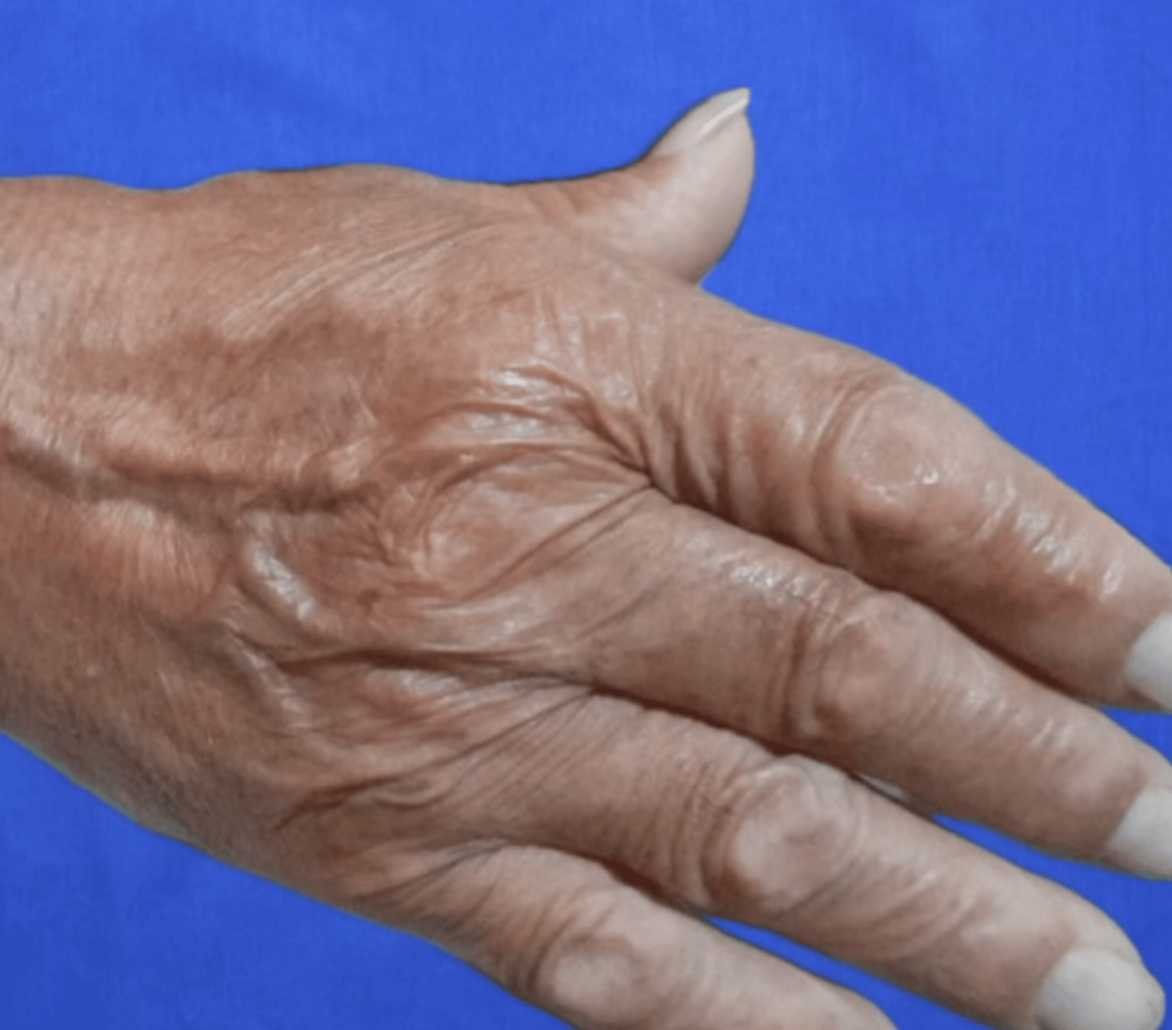
Annual follow-up image One-year follow-up image of the flap

At the one-year follow-up, the patient demonstrated good postoperative recovery, with satisfactory functional and aesthetic outcomes (Figure 5). Sensibility of the fingertip was preserved, with no neurological deficits reported. Active range of motion of the interphalangeal joints, including extension, was maintained without functional limitation. The germinal matrix and nail apparatus were unaffected by the injury, and no nail deformities were observed at follow-up.

## Discussion

The reverse-flow dorsal digital and metacarpal artery flaps are well-established reconstructive options for soft tissue defects of the phalanges. Their vascular basis lies in consistent anastomoses between the DMAs and the palmar digital system, which allow for reliable perfusion without compromising the volar digital artery [6,7]. These flaps are classified as homodigital, minimizing morbidity by preserving adjacent digits. They are particularly effective for dorsolateral and dorsal defects due to their wide arc of rotation and dependable skin paddle. In our case, the extended reverse DMA (RDMA) flap was successfully used in a 77-year-old agricultural worker with poorly controlled diabetes, highlighting its viability even in high-risk individuals. Previous reports have demonstrated that the RDMA flap can be elevated under local anesthesia in an outpatient setting, with minimal immobilization required postoperatively [7]. These characteristics are particularly advantageous in elderly or comorbid patients, where prolonged hospitalization and complex procedures are not ideal.

Although a full-thickness skin graft (FTSG) might be considered a lower morbidity option, it was not suitable in this case due to two main reasons. Firstly, the defect involved exposed extensor tendon and cortical bone, both of which provide poor vascular beds for graft take in the absence of periosteum or paratenon. Second, patient-specific factors significantly influenced the reconstructive decision. The patient was a rural worker with limited access to specialized care and was identified from the outset as having a low likelihood of strict postoperative follow-up and meticulous wound care - both prerequisites for graft survival. Under these conditions, a vascularized flap represented a more reliable and durable option.

However, despite its advantages, the RDMA flap carries certain drawbacks, such as the relative bulkiness of the pedicle and the risk of early venous congestion, typically occurring within the first postoperative week [8]. In our patient, partial distal flap loss of approximately 30% occurred but was manageable without reintervention, aligning with previous reports of increased flap vulnerability in the setting of vascular comorbidities [9]. Of note, even with partial necrosis, the vascularized tissue promoted secondary healing, ultimately yielding satisfactory functional and aesthetic outcomes at one year.

What distinguishes this case is the immediate use of the extended RDMA flap in the emergency setting, which allowed for prompt coverage of tendon- and bone-exposed tissue, preventing secondary infection and facilitating early functional recovery. This reinforces the flap’s value as a first-line reconstructive option, even in complex defects and in patients considered suboptimal surgical candidates. Overall, our findings are consistent with existing literature supporting the RDMA flap as a versatile and dependable reconstructive technique. While further studies are warranted to better define outcomes related to this technique in diabetic and elderly populations, this report adds to the growing body of evidence endorsing its utility, even under less-than-ideal patient circumstances.

## Conclusions

Over the years, dorsal middle phalanx injuries have been repaired using various techniques, some of which involved two-stage procedures or limited early limb mobilization. Reverse-flow metacarpal artery flaps have provided an effective tool, yielding long-term functional and aesthetic results with minimal complications, which are often preventable with proper technique or manageable when promptly addressed. Despite being considered a challenging case due to advanced age and comorbidities, our patient was able to return to daily activities early, without the need for rehabilitation, achieving excellent functional outcomes. Although venous congestion was observed, which might have been avoided if a skin bridge had not been preserved, the results at the annual follow-up were exceptional.
